# A Miniature Intermittent-Flow Respirometry System with a 3D-Printed, Palm-Sized Zebrafish Treadmill for Measuring Rest and Activity Metabolic Rates

**DOI:** 10.3390/s20185088

**Published:** 2020-09-07

**Authors:** Shih-Hao Huang, Chia-Wei Tsao, Yan-Hung Fang

**Affiliations:** Department of Mechanical and Mechatronic Engineering, National Taiwan Ocean University, Keelung 202-24, Taiwan; 10672019@mail.ntou.edu.tw (C.-W.T.); 10872026@mail.ntou.edu.tw (Y.-H.F.)

**Keywords:** zebrafish, swimming ability, intermittent-flow respirometry

## Abstract

Zebrafish are a preferred vertebrate model for evaluating metabolism during development, and for toxicity studies. However, commercially available intermittent-flow respirometry systems (IFRS) do not provide a suitable zebrafish-scaled swimming tunnel with a low water volume and proper flow velocities. We developed a miniature IFRS (mIFRS) with a 3D-printed, palm-sized zebrafish treadmill for measuring the swimming ability and metabolic rate of a single one- or three-month-old zebrafish with and without toxicity treatment. The 3D-printed zebrafish treadmill consists of discrete components assembled together which enables the provision of a temporary closed circulating water flow. The results showed that three-month-old zebrafish of normal physiological status had higher energetic efficiency and could swim at a higher critical swimming speed (U_crit_) of 16.79 cm/s with a lower cost of transport (COT_opt_) of 0.11 μmol g^−1^m^−1^. However, for a single three-month-old zebrafish treated with an antibacterial agent, U_crit_ decreased to 45% of normal zebrafish and the COT_opt_ increased to 0.24 μmol g^−1^m^−1^, due to the impairment of mitochondria. Our mIFRS provides a low-cost, portable, and readily adaptable tool for studying the swimming performance and energetic metabolism of zebrafish.

## 1. Introduction

Recently, the zebrafish (*Danio rerio*) has become an important vertebrate model organism, and is extensively used in studies into genetics, development, immunology, physiology, toxicology and aging [1]. As well as their small size, optical transparency of complex organs, and ease of culture, one of the most attractive features of zebrafish is their age-related swimming ability and aerobic metabolism [2,3]. After hatching from the chorion, zebrafish typically display short and intermittent bursts of high-speed propulsion. After seven days post fertilization (dpf), larval zebrafish start to exhibit spontaneous swimming activity, and gradually grow up to increase their swimming velocities [4].

To evaluate the swimming ability and metabolic rate of aquatic organisms in the g to kg range, such as salmon, tilapia, trout, and sturgeon, intermittent-flow respirometry systems (IFRS) with a liter-sized swimming tunnel are usually used [5,6,7,8,9,10]. The IFRS enables the provision of a small temporary circulating water flow with stepwise increasing flow velocities and allows measuring of oxygen consumption rates (OCRs) to subsequently estimate the aerobic metabolic rates of aquatic animals at a specific flow velocity. The standard metabolic rate (SMR) and maximum metabolic rate (MMR) are two fundamental physiological variables in aerobic energy metabolism. SMR stands for the minimal resting metabolic rate of an unstressed and normal physiological status, while MMR reflects the maximum capability for aerobic energy metabolism, which is conventionally measured at the maximum sustainable swimming speed with a maximal OCR. The aerobic metabolic scope (AMS), calculated by subtracting the SMR from the MMR, represents the total amount of aerobic energy available to the animal for energy-demanding processes [6].

However, the conventional IFRS with a liter-sized swimming tunnel does not provide a suitable zebrafish-scaled swimming tunnel with a low water volume and proper flow velocities to precisely measure the metabolic rates at a single zebrafish level because of the small size and relatively slow swimming speeds of zebrafish. Recently, the IFRS with a milliliter-sized swimming tunnel of a glass tube from Loligo Systems (www.loligosystems.com) have been typically used to study the swimming performance and active metabolic rates of larvae, juveniles and adult zebrafish in studies of endurance exercise [11,12], aerobic/hypoxic metabolism [13,14], and drug toxicity [15,16]. However, this miniature IFRS is expensive and the assembly of the whole system is also complicated and time-consuming. These measurements require more than three testing zebrafish simultaneously swimming in the swimming tunnel, in order to generate sufficient oxygen consumption to estimate aerobic metabolic rates. Because of hydrodynamic advantages from group swimming, the swimming ability and metabolic rates of individual zebrafish would be underestimated.

Instead, researchers often build a custom swimming tunnel or flume for their zebrafish studies [17,18,19,20,21]. Widrick et al. [20] developed an easily assembled, open-source zebrafish-scaled flume, made from a clear polycarbonate cylindrical tube with two independent spherical-impeller pumps modulated by a microcontroller to achieve flow velocities ranging from 1 to 70 cm/s for assessing the swimming performance of larval, juvenile, and adult zebrafish. Although their proposed flume is inexpensive, easily constructed, and readily adaptable, they did not integrate an oxygen sensor to perform oxygen consumption measurements and therefore permit the estimation of aerobic metabolic rates. Mwaffo et al. [21] fabricated a miniature swimming tunnel with a volume of 0.6 L, which enabled the analysis of the flow field around the zebrafish using particle image velocimetry (PIV) to explore the topology of vortex shedding for different patterns of zebrafish locomotion. They fabricated partial components of their miniature swimming tunnel using 3D-printed technology, such as funnels, a locker, and end caps, used to mount at the inlet and outlet of a 15 cm long acrylic tube with a 4.57 cm inner diameter, which formed the testing section for the zebrafish. Palstra et al. [17] fabricated a small Blazka-style swimming respirometer with a water volume of 1.8 L, which enabled swimming experiments on adult zebrafish to quantify the economy of swimming by measuring oxygen consumption and demonstrating the effects of exercise on swimming-enhanced growth and the expression of muscle growth marker genes in adult zebrafish. However, these measurements still require more than 10 adult zebrafish in the 1.8 L swimming tunnel, in order to generate sufficient oxygen consumption. The more adult zebrafish used, the greater the possibility of losing information about individual differences.

We developed a miniature intermittent flow respirometry system (mIFRS) with a 3D-printed, palm-sized zebrafish treadmill, for measuring the swimming ability and metabolic rate of a single one- or three-month-old zebrafish to evaluate their optimal swimming speeds and transport costs with and without toxicity treatment. The 3D-printed zebrafish treadmill with a 20 mL water volume consists of discrete components assembled together like modules, including a 3D-printed propeller to drive the flow, flow-rectified structures, honeycomb screen connectors for lamellar flow, a test section to accommodate a single zebrafish, and a fiber-optic oxygen sensing system to measure dissolved oxygen. The 3D-printed zebrafish treadmill enables the provision of temporary closed circulating water with a low water volume and proper flow velocities to precisely measure the metabolic rates at a single zebrafish level while performing intermittent flow respirometry. We have successfully demonstrated the use of our mIFRS to measure the critical swimming speed (U_crit_), optimal swimming speed (U_opt_), SMR, MMR, and cost of transport (COT) of individual one- and three-month-old zebrafish with and without exposure to the antibacterial agent (Triclosan).

## 2. Materials and Methods

### 2.1. Design of Miniature Intermittent-Flow Respirometry Systems (mIFRS)

**Figure 1:**
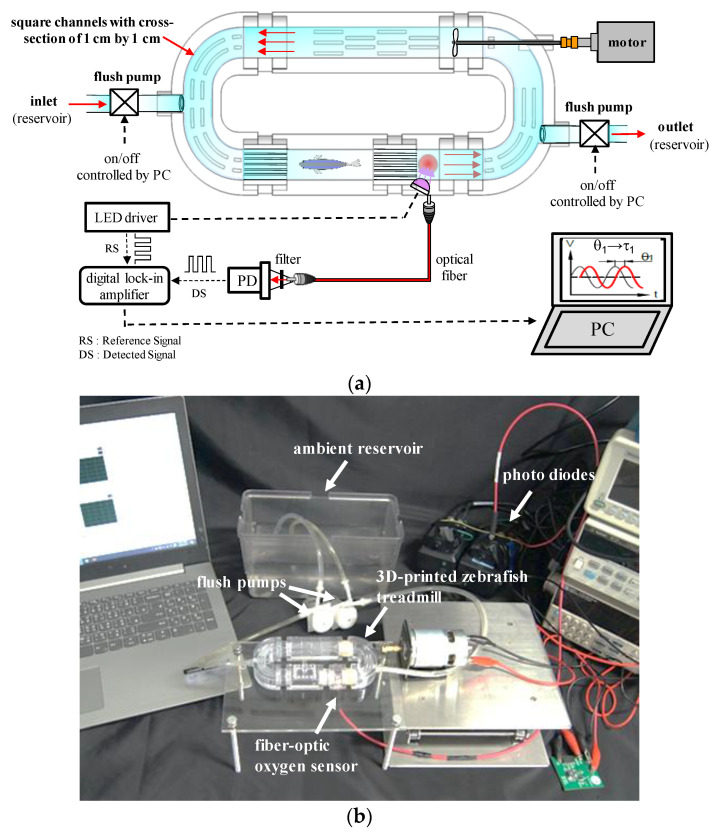
(**a**) Schematic and (**b**) image of the miniature intermittent flow respirometry system (mIFRS), composed of a 3D-printed, palm-sized zebrafish treadmill to provide a swimming tunnel, a fiber-optic oxygen sensing system to measure concentrations of dissolved oxygen, and a custom-made PC-based control system to automatically turn the flush pumps on and off to create a temporary closed circulating water flow. Intermittent-flow respirometry is a series of open and closed water circulation phases at a preset circulation water velocity with concomitant oxygen measurements. A single test zebrafish was placed into a 3D-printed zebrafish treadmill to evaluate its swimming ability, aerobic metabolic rates, optimal swimming speeds, and cost of transport by calculating the oxygen consumption rates of the test zebrafish.

Figure 1a,b show the schematic and image of the miniature intermittent flow respirometry system (mIFRS), composed of a 3D-printed, palm-sized zebrafish treadmill to provide a temporary closed circulating water flow, a fiber-optic oxygen sensing system to measure the concentrations of dissolved oxygen, and a custom-made PC-based control system to automatically turn the flush pumps on and off for automated intermittent-flow respirometry. Intermittent-flow respirometry is a series of open and closed water circulation phases at a preset circulation water velocity with concomitant oxygen measurements. The control system can automatically turn flush pumps on and off to create a temporary closed circulating water flow in the 3D-printed zebrafish treadmill for a preset time period for automated intermittent-flow respirometry.

#### 2.1.1. 3D-Printed, Palm-Sized Zebrafish Treadmill

**Figure 2:**
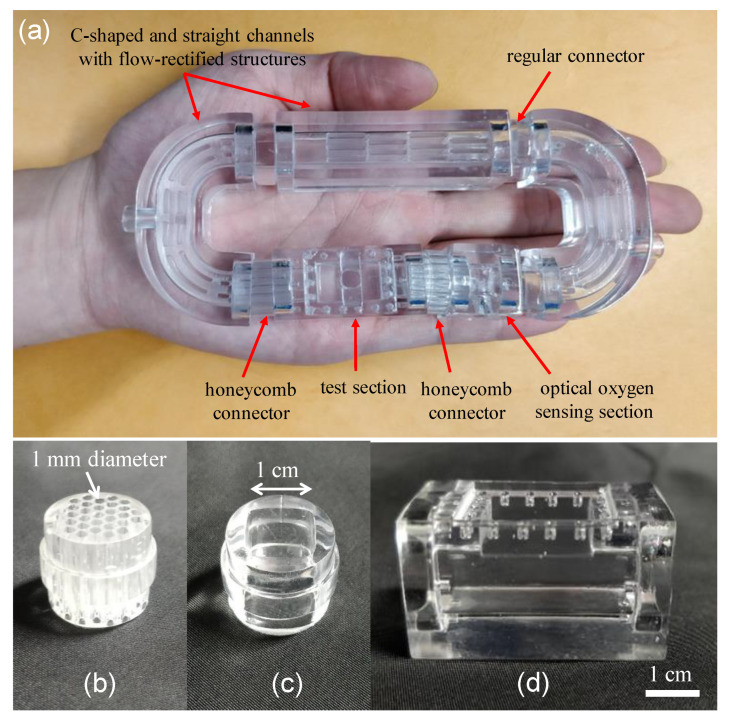
(**a**) An image of a 3D-printed, palm-sized zebrafish treadmill with a 20 mL water volume consisting of discrete components assembled together like modules. Images of (**b**) honeycomb screen connector, (**c**) regular connector and (**d**) a test section to accommodate a single zebrafish.

The 3D-printed zebrafish treadmill with a 20 mL water volume consists of discrete components, including two C-shaped channels and one straight channel with flow-rectified structures, honeycomb screen connectors for lamellar flow, a test section to accommodate a single zebrafish, and an optical oxygen-sensing section to measure dissolved oxygen (Figure 2a). The design concept of the 3D-printed zebrafish treadmill is similar to assembling modules, in that we can quickly modify an assembly or replace a component in order to optimize operation. All components were designed using computer-aided design (CAD) software and printed using UnionTech Lite 600 (Union Tech, Inc., St. Charles, IL, USA) and WaterShed XC 11,122 resin (DSM Somos^®^, Heerlen, Netherlands) in high-resolution mode. This nearly transparent resin provides well-characterized mechanical strength and good biocompatibility [22]. All components were designed with a square cross-sectional channel (1 cm × 1 cm) to ensure optical clarity through their interfaces, and each component is connected by a cylindrical regular connector or honeycomb screen connector. Figure 2b–d show images of the cylindrical honeycomb screen connector, regular connector, and a test section to accommodate a single zebrafish. Two honeycomb screen connectors, designed to have a cylindrical structure of 16 mm in diameter and 36 mm in length, containing 30 pores with a diameter of 1 mm, are connected at the inlet and outlet of the test section to form a lamellar flow and to confine the zebrafish within the test section during the measurements (Figure 2a). Regular connectors without honeycomb screen structures are used to connect the other components. The test section was designed to have a square cross-sectional channel (1 cm × 1 cm), 6 cm in length, which accommodates a single zebrafish, because three-month-old adult zebrafish rarely exceeded 0.5 g in mass and 3 cm in length. The test section was designed to have an opening at the top surface, which is sealed with a cover, to easily load and unload a single zebrafish.

**Figure 3:**
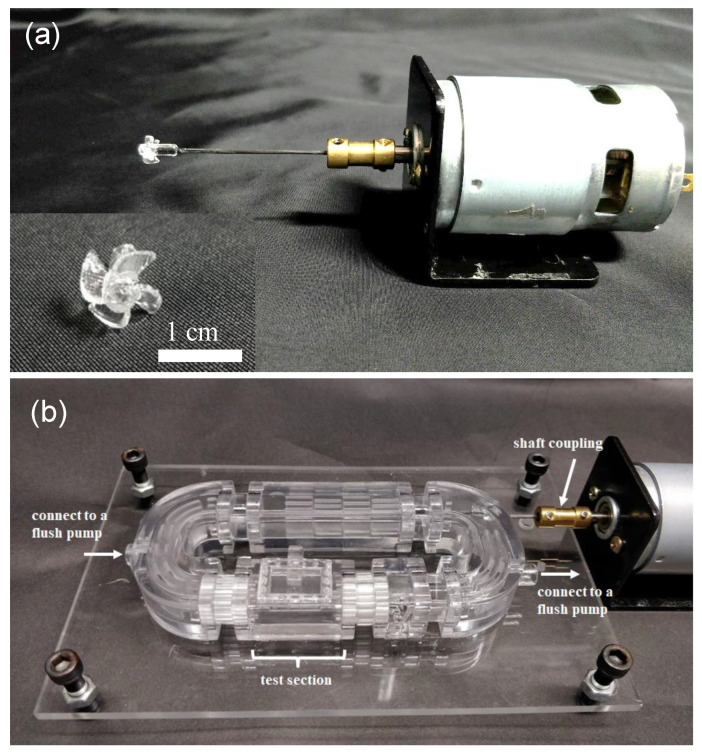
(**a**) Image of the 3D-printed 4 blade propeller, which was mounted on a 3 mm diameter shaft and connected to a DC motor via a shaft coupling. (**b**) The 3D-printed propeller was assembled into a 3D-printed zebrafish treadmill with a fixing structure at the C-shaped channel to support the shaft and avoid shaft vibration.

Figure 3a shows an image of the 3D-printed four-blade propeller with a diameter (D) of 8 mm, pitch (P) of 16 mm, and a 45° blade angle of attack, which is mounted on a 3 mm diameter shaft and connected to a DC motor via a shaft coupling. The diameter is the overall size of the blade as measured from the tips. A larger diameter propeller can push more water than a small diameter propeller, creating more power. The pitch is the distance at which the propeller will move the fluid after one revolution. A high-pitched propeller allows the flow to move faster by traveling further with each rotation. The ratio (P/D) typically falls between 0.5 and 2.5, with an optimal value for the most best-performing propeller close to 0.8 to 2.0 [23]. The ratio (P/D) of our propeller is 2.0. This 3D-printed propeller was then assembled into a 3D-printed zebrafish treadmill with a fixing structure at the C-shaped channel to support the shaft and avoid shaft vibration (Figure 3b).

#### 2.1.2. A Fiber-Optic Oxygen Sensing System

**Figure 4:**
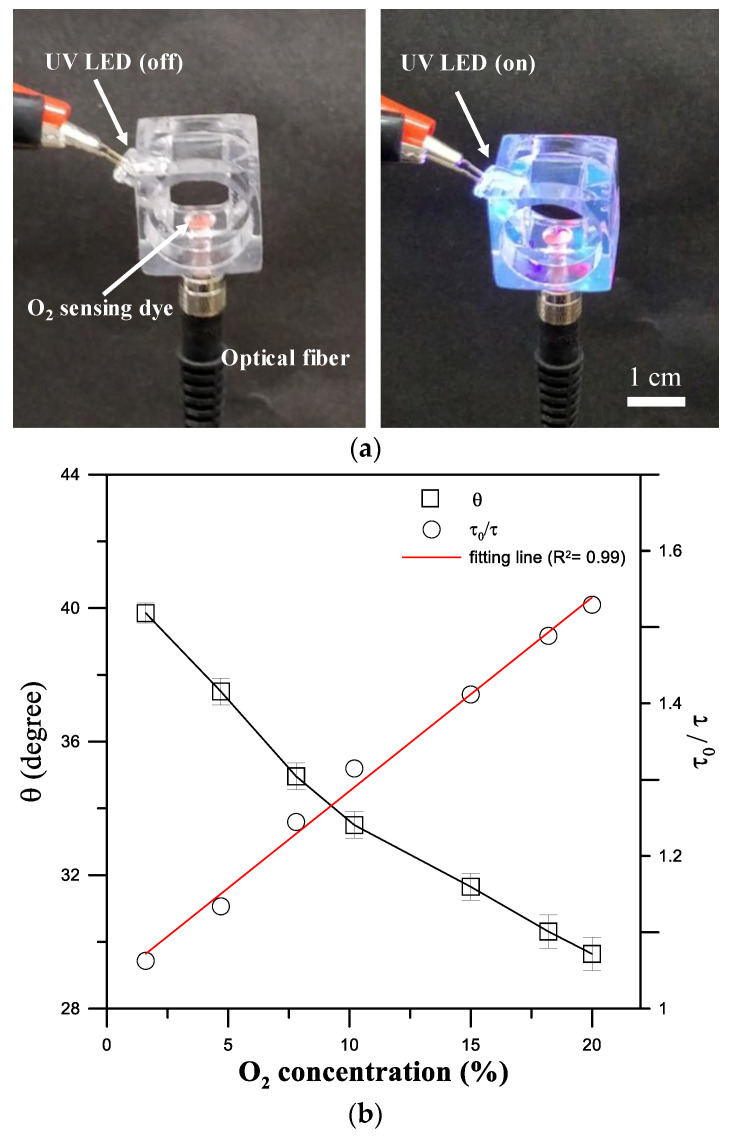
(**a**) A fiber-optic oxygen sensing section with a 1 mm diameter microwell coated with an oxygen-sensitive layer and excited by a UV LED modulated at 5 kHz to measure the luminescent phosphorescence lifetime of the oxygen-sensitive layer to quantify O_2_ concentrations. (**b**) Phase shift (*θ*) and normalized lifetime (*τ*_0_/*τ*) as a function of the dissolved oxygen (DO) concentration. (Mean ± SEM; *n* = 5).

A fiber-optic oxygen sensing system was developed to measure oxygen concentration changes based on the phase-based phosphorescence lifetime measurement during intermittent flow respirometry (Figure 1). Measuring the lifetime of luminescent phosphorescence to quantify oxygen concentration has been shown to have high sensitivity and stability, and is not susceptible to variations in intensity of incident light or the inhomogeneous distribution of an oxygen-sensitive layer [24]. The optical oxygen-sensing section (Figure 4a) was designed to have a 1 mm diameter microwell at the bottom of the channel, where Pt(II) octaethylporphyrin (PtOEP, λ_ex_ = 381 nm, λ_em_ = 646 nm, Sigma Aldrich, St. Louis, MO, USA) was deposited as an oxygen-sensitive luminescent layer within the microwell for O_2_ detection. PtOEP displays strong phosphorescence, with a long lifetime, and does not consume oxygen or generate toxic byproducts as part of the sensing process [25].

A 3 W high-power UV LED (390 nm, Edison Opto Corp., Taipei, Taiwan) modulated at a frequency of 5 kHz was set up as the light source at the side channel to excite the oxygen-sensitive luminescent layer (PtOEP). The light intensity of the phosphorescence emitted from the PtOEP sensing layer is collected via a multi-mode optical fiber (NA = 0.5, LED470L, Thorlabs Inc., Newton, NJ, USA) positioned below the PtOEP sensing layer and detected by an amplified photodetector (PDA100A, Thorlabs Inc., Newton, NJ, USA). A long-pass optical filter for isolating the background is involved in the coupling between the photodetector and optical fiber. The phase shift (*θ*) between the reference signal (RS, LED modulation light) and the detection signal (DS, the corresponding phosphorescence) is determined using digital lock-in analysis via a custom-made LabVIEW program (National Instruments Inc., Austin, TX, USA) (Figure 1). The details of the facility setup and data acquisition were described in our previous work [25,26,27]. The phase shift (*θ*) changes, as the oxygen concentration changes, is related to the phosphorescence lifetime (*τ*), as shown in Equation (1):

(1) tan(θ)=2πfτ,

where *f* is the modulation frequency of the UV LED light. The relationship between the luminescence intensity (*I*) and lifetime (*τ*) in the absence (*I*_0_, *τ*_0_) and presence (*I, τ*) of oxygen follow the Stern-Volmer equation as follows [24]:

(2) $$\frac{I_{0}}{I}=\frac{\tau_{0}}{\tau }=1+{Κ}_{SV}\left[O_{2}\right],$$

where *K_sv_* is the Stern-Volmer constant, and [O_2_] is the oxygen concentration in the solution. Calibration tests on the phase shift (*θ*) versus the dissolved oxygen (DO) concentration are performed by introducing 0–20% (i.e., 0–8.16 mg/L) DO concentrations into the swimming tunnel. Figure 4b shows the variation in the phase shift (*θ*) and the corresponding normalized lifetime (*τ*_0_/*τ*) as a function of the DO concentration measured at a modulated excitation light at 5 kHz. A nearly linear relationship was observed between the normalized lifetime (*τ*_0_/*τ*) and the DO concentration (R^2^ = 0.99).

#### 2.1.3. A Custom-Made PC-Based Control System

A custom-made PC-based control system was developed to drive the digital relay (USB-SwitchC IEC, Cleware Inc., Hollingstedt, Germany). The control system can automatically turn flush pumps (50 mL/min, CS074-3S, C-BLUE Inc., Kaohsiung, Taiwan) on and off to create a temporary closed circulating water flow in the 3D-printed zebrafish treadmill for a preset time period for automated intermittent-flow respirometry. One complete measurement cycle of the intermittent-flow respirometry consists of three time periods: the flush (t_f_), wait (t_w_), and measurement (t_m_) phases. At the flush phase (t_f_), denoted as the F-phase, the flush pumps are switched on by a digital relay to replenish the 3D-printed zebrafish treadmill with fresh water via the ambient reservoir. In the wait phase (t_w_), denoted the W-phase, the flush pumps are switched off and the 3D-printed propeller is constantly driven to provide a temporary closed circulating water flow inside the 3D-printed zebrafish treadmill. During the measurement phase (t_m_), denoted the M-phase, the oxygen level is continuously measured by a fiber-optic oxygen sensing system. The time for each phase (t_f_, t_w_, and t_m_) was set to t_f_ = 5 min, tw = 3 min, and tm = 5 min, unless otherwise stated. The length of the three phases can be optimized to match the experimental conditions. Three-phase operation for replenishing the 3D-printed zebrafish treadmill with fresh water (F-stage), closing the 3D-printed zebrafish treadmill to circulate the flow (W-stage), and measuring the oxygen concentration (M-stage) were repeated to continuously measure the variation in oxygen concentration within the 3D-printed zebrafish treadmill.

The metabolic rate (MO_2_) of a single zebrafish in a temporary closed circulating water flow was calculated from the decreased rate of oxygen concentration (Δ[O_2_]/Δt) during the measurement phase (t_m_) using open-source software (FishResp) [28]:

Metabolic rate (μmol h^−1^g^−1^): MO_2_ = V/m × Δ[O_2_]/Δt (3)

where Δ[O_2_]/Δt is the slope in μmol/L/h, V is the water volume of the swimming tunnel in L, and m is the mass of the zebrafish in g. FishResp is a user-friendly software with a graphical user interface (GUI) for analyzing raw data from intermittent-flow respirometry systems.

### 2.2. Animals and Housing

A total of 60 wild-type and AB strain zebrafish (*Danio rerio*) at 30 dpf (larval stage) and 90 dpf (young adult stage) were purchased from the G.fish Animal Model Co. (Taipei, Taiwan) for the experiments. The zebrafish were housed in stand-alone aquaria with a recirculating system, and kept in a 14 h light/10 h dark cycle, with the culture temperature maintained at 28 °C, according to standard methods [29]. The average body length and weight of the 30 dpf and 90 dpf zebrafish were 14.2 ± 0.5 mm, 24.8 ± 6 mg and 25 ± 1.0 mm, 121.1 ± 11.6 mg, respectively, as shown in Table 1. At least five replicate trials were performed in our experimental group. Individual one- and three-month-old zebrafish were randomly selected from 60 zebrafish to account for possible individual differences.

### 2.3. Swimming Performance Protocol

**Figure 5:**
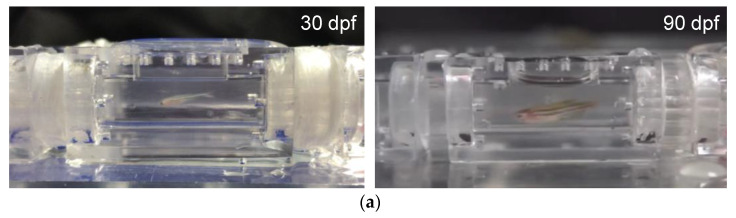

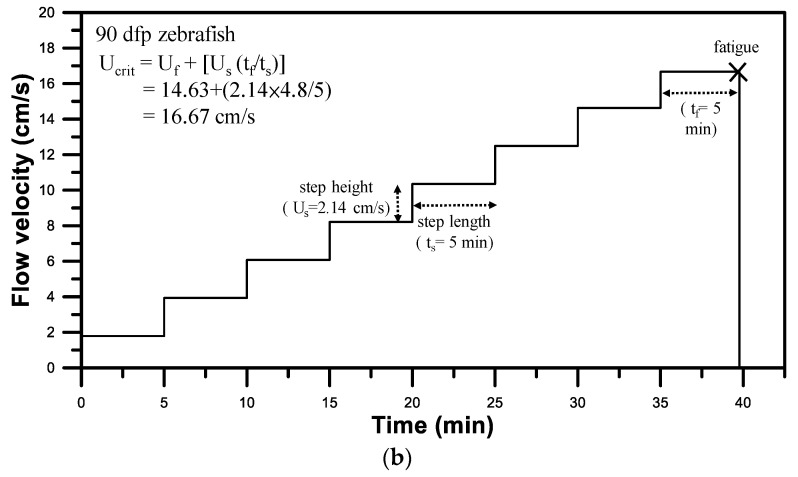
(**a**) Images of 30 dpf larval zebrafish and 90 dpf young adult zebrafish swimming in the test section of the 3D-printed zebrafish treadmill against a water flow of 35% U_crit_ (Supplementary Videos S1 and S2). (**b**) Swimming performance protocol used in swimming assessments of 90 dpf zebrafish to evaluate the U_crit_ to be U_crit_ = 16.67 cm/s.

A 20 mL 3D-printed zebrafish treadmill was used to evaluate the swimming ability of a single zebrafish following the standard swimming performance protocol as described previously [2]. The test zebrafish were kept unfed for 24 h before being used for the swimming experiments. A single 30 dpf or 90 dpf zebrafish was placed in the test section of the 3D-printed zebrafish treadmill, where the zebrafish were forced to swim in the circulating water flow and recorded by a high-speed video camera (Figure 5a). The circulating water flow was generated by spinning a 3D-printed propeller. The velocity of the circulating water flow was initially set at U_w_ cm/s for 5 min for warming up, and then water velocity increased U_s_ cm/s every t_s_ min in a stepwise manner until the zebrafish became fatigued and could not keep swimming at a certain water velocity, as shown in Figure 5b. The critical swimming speed (U_crit_) of the testing zebrafish was calculated according to the following equation:

U_crit_ = U_f_ + [U_s_ (t_f_/t_s_)] (cm/s) (4)

where U_f_ is the water velocity of the last step completed before fatigue, U_s_ is the velocity increment at each step, t_f_ is the elapsed time the fish swims before fatigue, and t_s_ is the interval time of a step. For a 90 dpf zebrafish (young adult), the water velocity and time period were set at U_w_ = 1.8 cm/s for warming up with U_s_ = 2.14 cm/s and t_s_ = 5 min, as shown in Figure 5b, whereas for 30 dpf zebrafish (larvae), U_w_ = 0.72 cm/s, U_s_ = 0.31 cm/s, and t_s_ = 5 min. The water velocity was calibrated based on PIV analysis [21]. Fluorescent beads were introduced into the 3D-printed zebrafish treadmill, 2 to 9 V were applied to the DC motor with the 3D-printed propeller, and the movement of fluorescent beads was recorded using a high-speed video camera. The water velocities at the preset voltages were calculated using open-source PIV analysis software (OpenPIV) [30]. The water velocity showed a nearly linear relationship to the applied voltage (Supplementary Figure S1), which enabled the provision of a temporary closed circulating water flow with flow velocities ranging from 0.1 to 20 cm/s.

### 2.4. Cost of Transport (COT)

Automated intermittent flow respirometry was performed to measure the MO_2_ by calculating the oxygen consumption rates of the test zebrafish swimming at a minimal flow of 5% U_crit_, where the MO_2_ is the metabolic rate of the zebrafish used for routine swimming activity. Subsequently, the MO_2_ was measured at 35, 70, and 100% U_crit_. To estimate the SMR of zebrafish, the resulting MO_2_ (in μmol g^−1^h^−1^) was plotted against swimming speed U (in cm/s), which was fitted by the polynomial equation MO_2_ = SMR + aU + bU^2^ to estimate the SMR by extrapolating to zero (U = 0). In addition, the cost of transport (COT; μmol g^−1^m^−1^) was determined by dividing the MO_2_ values by the corresponding U values as follows [17]:

COT = MO_2/_U (μmol g^−1^m^−1^) (5)

The COT quantifies the energy efficiency of zebrafish by swimming a certain unit of distance at the corresponding velocity (U). A lower COT indicates higher energetic efficiency. The relationship between the COT and swimming speed U was plotted and fitted by the polynomial equation COT = a + bU + cU^2^, where the first derivative equals zero (i.e., dCOT/dU = 0) to calculate the U value, denoted as U_opt_. The COT at U_opt_ corresponds to the lowest COT, denoted as COT_opt_. U_opt_ indicates the speed at which the lowest COT occurred with the lowest oxygen consumption per unit distance swum.

## 3. Results and Discussion

### 3.1. Swimming Ability and Energetic Metabolism of a Single Zebrafish in the mIFRS

To demonstrate the ability of our mIFRS to study the swimming performance and energetic metabolism of zebrafish, individual one-month-old (30 dpf) larval zebrafish and three-month-old (90 dpf) young adult zebrafish were chosen due to the compatibility of their body size with the testing section, and their spontaneous and stable swimming activity. A single test zebrafish was placed into a 3D-printed zebrafish treadmill to evaluate its swimming ability, aerobic metabolic rates while swimming, optimal swimming speeds, and cost of transport. Figure 5a shows images of 30 dpf larval and 90 dpf young adult zebrafish swimming in the test section of the 3D-printed zebrafish treadmill against a water flow of 35% U_crit_ (Supplementary Videos S1 and S2). The 30 and 90 dpf zebrafish were able to swim at a U_crit_ of 3.75 cm/s and 16.79 cm/s (Table 1), respectively, after water velocities were gradually increased in a stepwise manner (Figure 5b). A young adult zebrafish could swim at a higher critical swimming speed than a larval zebrafish, indicating their age-related differences in their swimming ability [2,20].

**Figure 6:**
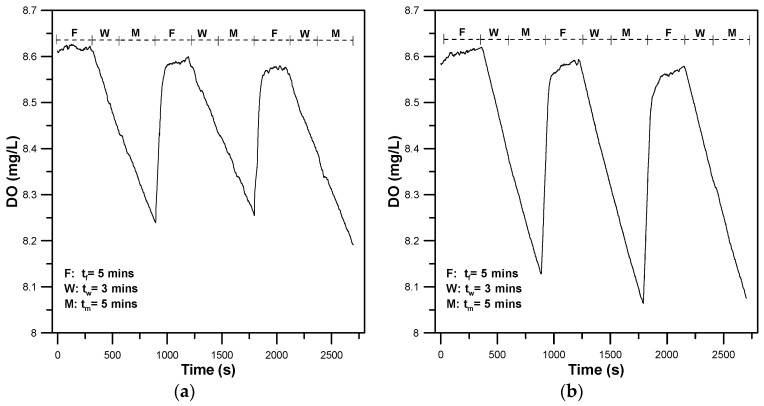
Representative results of the aqueous oxygen concentration (DO) over time for a single 30 dpf larval zebrafish swimming at the water flow of (**a**) 1.28 cm/s, and (**b**) 3.85 cm/s, using automated intermittent-flow respirometry.

To further assess the energy efficiency of 30 dpf larval and 90 dpf young adult zebrafish while swimming, automated intermittent-flow respirometry was performed to quantify their MO_2_ by measuring oxygen consumption to evaluate the aerobic metabolic rates, optimal swimming speeds (U_opt_), and cost of transport. Figure 6a,b show representative results of the aqueous oxygen concentration over time for a single 30 dpf larval zebrafish swimming at a water flow of 1.28 cm/s and 3.85 cm/s, respectively, by performing automated intermittent-flow respirometry. During the F-phase, the flush pumps were switched on for 5 min by a digital relay to replenish the 3D-printed zebrafish treadmill with fresh water via the ambient reservoir, to restore the zebrafish to normal status. The aqueous oxygen concentration was maintained at an approximately constant value of 8.6 mg/L over time. During the wait (3 min) and measurement (5 min) phases, as the flush pumps were switched off, the oxygen level inside the closed zebrafish treadmill started declining over time in a linear fashion. A short wait period (W-phase) was needed to allow the zebrafish to adapt itself to the new conditions in the closed zebrafish treadmill before the measurement period. The slope of the linear decline was used in Equation (3) to calculate the metabolic rate. As shown in Figure 6, the declined slope of the zebrafish swimming at U = 3.85 cm/s was larger than that of the zebrafish swimming at U = 1.28 cm/s, (i.e., larger MO_2_ needed at U = 3.85 cm/s). The flush phase was repeated after the measurement phase. At the F-stage, the two flush pumps enabled replenishment of the 3D-printed zebrafish treadmill with fresh water from an ambient reservoir, to rapidly restore the DO to approximately 8.6 mg/L within 30 s. Using a custom-made PC-based control system that can automatically turn flush pumps on and off, we performed three measurements, by periodically flushing the 3D-printed zebrafish treadmill. Each measurement period involved a three-phase operation to replenish the 3D-printed zebrafish treadmill with fresh water, closing the 3D-printed zebrafish treadmill to circulate the flow, and measuring the oxygen concentration. Blank tests without zebrafish were run at the end of each swim test to correct the oxygen dissolution in water due to the flow driven by the propeller and the contribution of oxygen consumption by other substances in water.

**Figure 7:**
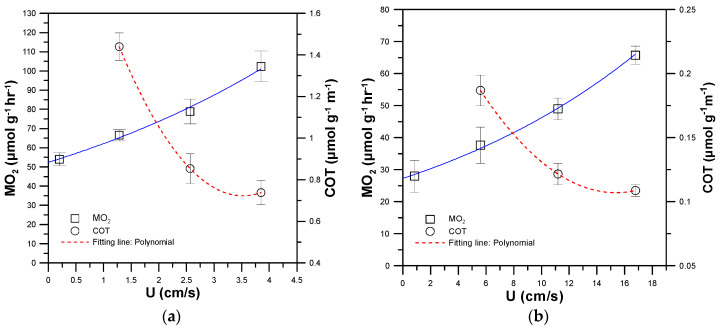
The metabolic rate (MO_2_) (white squares and solid blue line) and COT (white circles and broken red line) of single (**a**) 30 and (**b**) 90 dpf zebrafish swimming at 5, 35, 70 and 100% U_crit_, respectively. (Mean ± SEM; *n* = 9).

Figure 7a,b show the MO_2_ (white squares and solid blue line) and COT (white circles and broken red line) of single 30 and 90 dpf zebrafish swimming at 5, 35, 70, and 100% U_crit_, respectively. The COT of zebrafish swimming at 5% U_crit_ was omitted due to its relatively larger value when dividing MO_2_ by the corresponding U close to zero. The 30 and 90 dpf zebrafish were able to swim at U_crit_ of 3.75 cm/s and 16.79 cm/s, respectively. By fitting the MO_2_ data using the polynomial equation and extrapolating to zero (U = 0), the SMR, MMR and AMS of the single 30 and 90 dpf zebrafish were calculated as 52.81, 102.32 and 49.51 μmol h^−1^g^−1^ and 26.55, 65.89 and 39.15 μmol h^−1^g^−1^, respectively (Table 1). The younger 30 dpf zebrafish possessed higher SMR, AMR and AMS, which can be attributed to the allometric relationship between metabolic rate (in μmol h^−1^g^−1^) and body mass (M, in g) in zebrafish with SMR = 8.5 M^−0.035^ and MMR = 25 M^−0.074^ [3]. The allometric relationship indicates that older zebrafish with larger body mass consume less oxygen per unit mass per unit time in their SMR and MMR. Ignoring the factor of body mass, older zebrafish still consume more oxygen per unit time (μmol h^−1^) than younger zebrafish.

By fitting the COT data by the polynomial equation and setting dCOT/dU = 0, the U_opt_ and COT_opt_ of the single 30 and 90 dpf zebrafish could be calculated to be 3.52 cm/s, 0.72 μmol g^−1^m^−1^, and 15.32 cm/s, 0.11 μmol g^−1^m^−1^, respectively (Table 1). The U_opt_ at which the energetic efficiency was highest corresponds to about 90% of U_crit_ for both 30 and 90 dpf zebrafish, a finding which is similar to those of previous work [17]. For zebrafish swimming at speeds lower than U_opt_, partial energy was lost due to inefficient energetic transfer, while at speeds higher than U_opt_, anaerobic metabolism dominates the energetic production, increasing lactate levels and causing earlier fatigue. A lower COT indicates higher energetic efficiency. In contrast with a 30 dpf zebrafish, the older 90 dpf zebrafish showed a higher energetic efficiency, and were able to swim at a higher critical swimming speed (U_crit_) of 16.79 cm s^−1^ and a higher optimal swimming speed (U_opt_) of 15.32 cm/s, with a lower cost of transport (COT_opt_) of 0.11 μmol g^−1^m^−1^. Although the older 90 dpf zebrafish possessed lower SMR, MMR, and AMS used for energy production, their higher energetic efficiency enabled them to have better swimming ability than the younger 30 dpf zebrafish.

### 3.2. Toxicity Assay of a Single Zebrafish with an Antibacterial Agent

To demonstrate the utility of our mIFRS for toxicity assays on the swimming performance and energetic metabolism in zebrafish, a single 90 dpf zebrafish was treated with an antibacterial agent (Triclosan, Sigma-Aldrich, St. Louis, MO, USA) and assessed using critical swimming speed (U_crit_) and COT as indicators. Triclosan (5-chloro-2-(2,4-dichlorophenoxy)phenol) is an antibacterial agent used in many consumer products, such as soaps, deodorants, mouthwashes, and toothpastes. Chronic exposure of zebrafish to Triclosan has been found to cause delayed development, malformations, delayed hatching, and inhibition of normal growth [31]. Zebrafish (90 dpf) were exposed to 5 μM Triclosan for 30 min and then placed into the 3D-printed zebrafish treadmill for measurements. The exposure time and concentration over 30 min and 5 μM in Triclosan used for treatment of 90 dpf zebrafish causes significant lethality.

**Figure 8:**
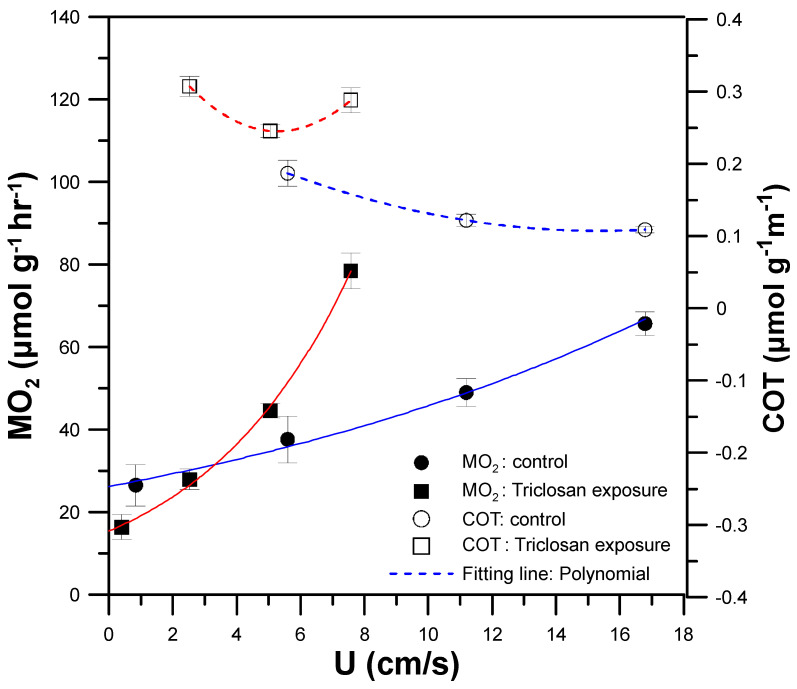
The metabolic rate (MO_2_) (black squares and circles with solid lines) and COT (white squares and circles with broken lines) of a single 90 dpf zebrafish swimming at 5, 35, 70 and 100% U_crit_ with or without (control group, normal zebrafish) exposure to 5 μM Triclosan for 30 min. (Mean ± SEM; *n* = 8).

Figure 8 shows the MO_2_ (black squares and circles with solid lines) and COT (white squares and circles with broken lines) of a single 90 dpf zebrafish swimming at 5, 35, 70, and 100% U_crit_ with or without (control group, normal zebrafish) exposure to 5 μM Triclosan for 30 min. There was a significant decrease in U_crit_ and U_opt_ to 7.57 cm/s (45% U_crit_ of normal zebrafish, control group) and 5.28 cm/s (34% U_opt_ of normal zebrafish, control group) (Table 1). In addition, the COT values of the zebrafish with Triclosan treatment (white squares with broken red lines) were larger than those of the normal zebrafish (white circles with broken blue lines). As mentioned above, a higher COT indicates a lower energetic efficiency. These results are attributed to the impairment of mitochondria in the zebrafish after Triclosan exposure. Triclosan has been shown to function as a mitochondrial uncoupler, and can impair mitochondrial functionality in zebrafish, causing inefficient production of adenosine triphosphate (ATP) [31], the energy currency of living cells. Inefficient ATP production lowers the energetic and swimming efficiency, which results in a decrease in U_crit_, along with an increase in COT values. Triclosan is also known to cause significant inhibition of acetylcholinesterase in the brain and skeletal muscle [32], which can degrade neuromuscular functionality and decrease U_crit_. Because of the lower energetic efficiency of zebrafish after Triclosan exposure, zebrafish require more oxygen to produce more ATP to drive swimming at any given speed, leading to a higher MMR (78.50 μmol h^−1^g^−1^) and producing fatigue at a lower final speed (U_crit_ = 7.57 cm/s), compared to normal zebrafish (control group) with MMR = 65.89 μmol h^−1^g^−1^ at U_crit_ = 16.79 cm/s. By using our mIFRS for the toxicity assay, we showed that the exposure of zebrafish to Triclosan had a significant effect on their swimming performance and energetic metabolism, decreasing U_crit_ and increasing COT. Further research is needed to understand the interaction between respiratory metabolism, energetic efficiency, and malfunction of mitochondria after Triclosan exposure, but are out of the scope of this study.

## 4. Conclusions

For the first time, we developed a mIFRS with a 3D-printed, palm-sized zebrafish treadmill to assess the resting and active metabolic rates of a single zebrafish by measuring oxygen consumption. On the basis of a single-zebrafish level, we can correctly measure the critical swimming speed and metabolic rates of individual zebrafish without overestimate/underestimate due to individual variations in swimming capacities. In addition, the 3D-printed zebrafish treadmill, which consists of discrete components fabricated by a 3D-printer, can be quickly assembled and replaced in order to optimize operation. We successfully demonstrated the ability of our mIFRS to study the swimming performance and energetic metabolism of individual one-month-old larval zebrafish and three-month-old young adult zebrafish with and without toxicity treatment. Although three-month-old zebrafish showed a higher energetic efficiency than one-month-old zebrafish, we still do not know when the energetic efficiency begins to decrease due to aging. By using our mIFRS, we will continue to investigate the effectiveness of exercise as a method to mitigate the effects of aging in zebrafish. Our mIFRS provides a low-cost, portable, and readily adaptable tool for studying the swimming performance and energy metabolism of zebrafish for applications in exercise physiology, toxicology, environmental science, and ecology.

## Figures and Tables

**Table 1:** Swimming performance and energetic metabolism of 30 and 90 dpf zebrafish for standard metabolic rate (SMR), maximum metabolic rate (MMR), critical swimming speed (U_crit_), optimal swimming speed (U_opt_) and optimal cost of transport (COT_opt_) at (U_opt_) with and without Triclosan exposure.

	Normal	Normal	Triclosan Exposure
**N**	9	8	8
**dpf**	30	90	90
**Mass** (mg)	24.8 ± 6	121.1 ± 11.6	128.05 ± 16.9
**Body length** (mm)	14.2 ± 0.5	25 ± 1.0	25.5 ± 1.5
**SMR** (μmol h^−1^g^−1^)	52.81	26.55	16.38
**MMR** (μmol h^−1^g^−1^)	102.32	65.89	78.50
**U_crit_** (cm/s)	3.85	16.79	7.57
**U_opt_** (cm/s)	3.52	15.32	5.28
**COT_opt_** (μmol g^−1^ m^−1^)	0.72	0.11	0.24

## Supplementary Materials

The following are available online at https://www.mdpi.com/1424-8220/20/18/5088/s1, Videos S1 and S2 show 30 dpf larval zebrafish and 90 dpf young adult zebrafish swimming in the test section of the 3D-printed zebrafish treadmill against a water flow of 35% U_crit_. Figure S1 shows the dependence of the water velocity and the applied voltages on a DC motor. All authors have read and agreed to the published version of the manuscript.
