# Supplementary material for: A Miniature Intermittent-Flow Respirometry System with a 3D-Printed, Palm-Sized Zebrafish Treadmill for Measuring Rest and Activity Metabolic Rates
Source: Sensors (Basel). 2020 Sep 7;20(18):5088. doi: 10.3390/s20185088 (PMC7570584; doi:10.3390/s20185088)
Supplement: Supplementary file 1 [file sensors-20-05088-s001.zip › Figure 1S.docx]

Figure S1. The dependence of the water velocity and the applied voltages on a DC motor.
